# Spine Alignment in Standing and Maximal Upper Limb Elevation in Baseball Players with Lumbar Spondylolysis and Those without Low Back Pain

**DOI:** 10.3390/ijerph20043231

**Published:** 2023-02-12

**Authors:** Kanta Matsuzawa, Tomoyuki Matsui, Yoshikazu Azuma, Tetsuya Miyazaki, Machiko Hiramoto, Ruo Hashimoto, Noriyuki Kida, Toru Morihara

**Affiliations:** 1 Marutamachi Rehabilitation Clinic, Kyoto 604-8405, Japan; 2 Department of Biotechnology, Graduate School of Science and Technology, Kyoto Institute of Technology, Kyoto 606-0951, Japan

**Keywords:** lumbar spondylolysis, lumbar lordosis angle, sacral slope angle, thoracic kyphosis angle, baseball

## Abstract

The changes in lumbar lordosis angle (LL) and sacral slope angle (SS) related to upper limb elevation and thoracic kyphosis angle (TK) in baseball players with spondylolysis remain unclear. Herein, we investigated baseball players with spondylolysis and those without low back pain, comparing LL and SS with upper limb elevation within and between groups and TK between groups. Baseball players with spondylolysis were enrolled as subjects, and baseball players without low back pain were enrolled as controls (n = 8 each). X-rays were obtained in the standing position and with maximal elevation position of the upper limb (elevation position). LL and SS were measured in the standing and elevated positions, and TK was measured in the standing position. LL was significantly larger in individuals with spondylolysis than controls. The SS of the control group was significantly larger in the elevated position than in the standing position, while the SS of the spondylolysis group was not significantly different between positions. SS was significantly larger in the spondylolysis group than in the control group, only in the standing position. Physical therapy for spondylolysis should focus on hyperlordosis alignment in the standing and maximal elevation positions of both upper limbs, sacral hyper-slope alignment in the standing position, and decreased sacral slope motion.

## 1. Introduction

Low back pain is common in growing baseball players, with lumbar spondylolysis being its common cause [1]. Spondylolysis is a stress fracture of the pars interarticularis [2] and is commonly managed with conservative treatment [3,4]. However, the recurrence rate is high, at 26.1% [5], and there is a risk of transition to isthmic spondylolisthesis [6]. Improving physical function through physical therapy is considered important to prevent this [6,7,8]. There have been several reports on the characteristics of physical function in spondylolysis patients [8,9,10,11,12]; however, to date, none have examined the characteristics of patients who are baseball players.

A previous study reported that the lumbar lordosis angle (LL) during standing was higher in spondylolysis patients than healthy subjects [9]. An increase in LL may lead to an increase in the forces on the pars interarticularis with lumbar extension [13]. Additionally, the sacral slope angle (SS) can indicate the risk of progression from spondylolysis to spondylolisthesis [14]. A previous study [8] reported no significant difference in SS between patients with spondylolysis and those with low back pain not caused by spondylolysis. However, we believe that assessment related to the upper limb elevation is important, since baseball is an overhead sport in which the maximum shoulder abduction is about 100° (80–120°) when pitching [15]. It has been reported that upper limb elevation requires lumbar extension [15]. In patients with spondylolysis, an excessive increase in LL or SS with upper limb elevation may negatively affect prognosis. In addition, an increase in the thoracic kyphosis angle (TK) in standing position limits upper limb elevation [16], and compensatory lumbar extension may occur in such cases. Moreover, because the stresses applied to the pars interarticularis of the lumbar spine during extension are high [17], we believe it is important to examine the TK characteristics of spondylolysis. To the best of our knowledge, there exists a lack of documentation regarding changes in LL and SS related to upper limb elevation and TK among spondylolysis patients. Clarifying these findings may help in the physical therapy of patients with spondylolysis.

The purpose of this study was to compare LL and SS with upper limb elevation within and between groups of baseball players with spondylolysis and those without low back pain, as well as to compare TK between the groups. We hypothesized that TK, LL, and SS would increase during standing in baseball players with spondylolysis compared to those without low back pain and that LL and SS would increase with upper limb elevation only in patients with spondylolysis.

## 2. Subjects and Methods

### 2.1. Subjects

This was a retrospective study. The participants comprised eight young male baseball players with lumbar spondylolysis who visited our clinic for an initial visit between January 2021 and December 2022 (mean age, 14.8 ± 2.2 years; 7 at L5, 1 at L3; height, 164.0 ± 11.9 cm; body mass, 60.6 ± 16.9 kg). A control group of eight age-matched male baseball players with throwing elbow injuries and without low back pain in life and sports who visited our clinic during the same period were enrolled as controls (mean age, 14.8 ± 0.7 years; height, 166.0 ± 3.2 cm; body mass, 61.8 ± 9.2 kg). The 16 participants were Japanese. In our clinic, lateral X-ray films were usually taken of patients with low back pain and throwing elbow injuries in the standing and upper limb maximum elevation. The exclusion criteria were as follows: patients with a history of orthopedic surgery, shoulder pain, and in whom radiographs were not taken in the standing position and during maximum upper limb elevation.

In a previous study [9], the standard deviations of LL and SS were 13° and 10°, respectively. Assuming a change of 5° in this study, we set the effect size at 0.5. Using G*Power, we calculated the sample size based on the interaction of two-way analysis of variance (ANOVA; α error of 0.05, and power of 0.8), yielding a required sample size of 8. Accordingly, we enrolled a total of 16 participants who met this requirement. This study was performed in accordance with the Declaration of Helsinki after obtaining approval from the ethics committee at our institution (Raku-gaku-Rin-01-000100).

### 2.2. Measurements

**Figure 1:**
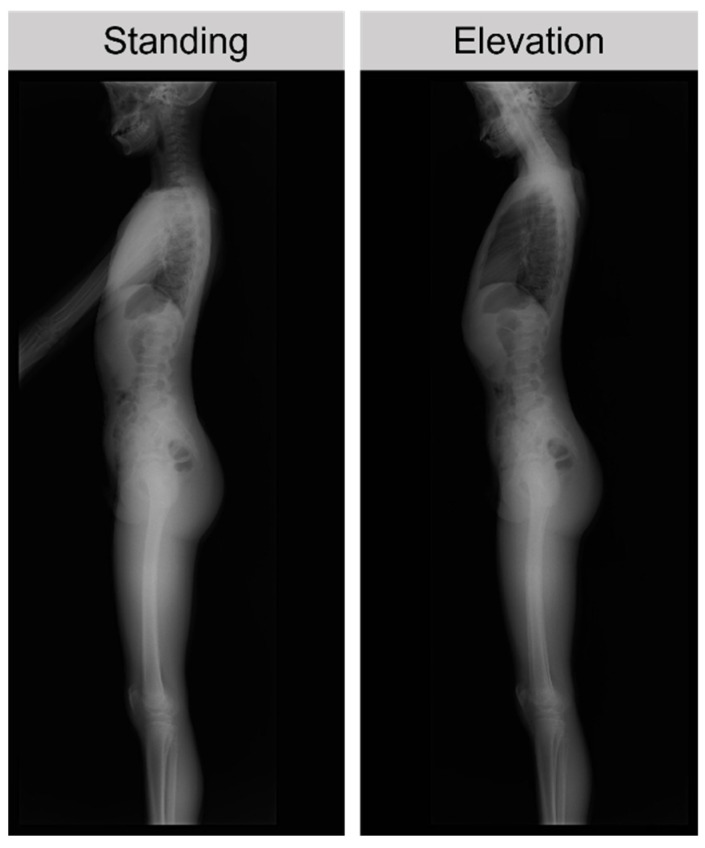
Patient position for X-ray evaluation. The patient was instructed to elevate both upper limbs to the maximum during elevation.

X-ray films were taken in the standing position and with upper limb elevation (elevation position) (Figure 1). During elevation, the patient was instructed to elevate both upper limbs to the maximum. The LL and SS were measured using two types of X-ray films. TK was measured using standing X-ray films.

**Figure 2:**
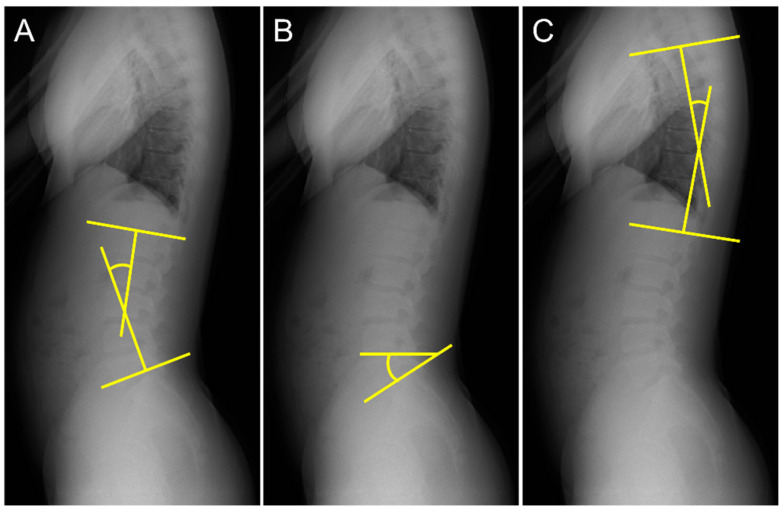
Measurement of spinal alignment. (**A**) Measurement of the lumbar lordosis angle (LL), (**B**) sacral slope angle (SS), and (**C**) thoracic kyphosis angle (TK).

The lumbar lordosis angle (LL) was measured according to the method outlined in a previous study [18,19]. First, lines were drawn tangential to the superior endplate of L1 and inferior endplate of the L5. Second, perpendicular lines were drawn to each tangent. LL was defined as the acute angle formed by the intersection of the two perpendicular lines (Figure 2A). The sacral slope angle (SS) was measured using the procedure described in a previous study [20]. Lines were drawn tangentially to the superior endplate of S1 and horizontally. The SS was defined as the acute angle formed by these two lines (Figure 2B). The thoracic kyphosis angle (TK) was measured using the procedure described in a previous study [20]. First, lines were drawn tangential to the superior endplate of T5 and inferior endplate of T12. Second, lines perpendicular to each tangent were drawn. TK was defined as the acute angle formed by the intersection of the two perpendicular lines (Figure 2C).

### 2.3. Statistical Analysis

The data distribution was assessed using a Shapiro–Wilk test, and we confirmed that the data followed a normal distribution. Split-plot ANOVA (group factor (spondylolysis, control) × position factor (standing, elevation position)) was performed for LL and SS. When the interaction was significant, a simple main effect was examined using Bonferroni’s method. Values of partial η^2^ of 0.0099, 0.0588, and 0.1379 corresponded to small, medium, and large effects, respectively [21]. Student’s *t*-test was performed to compare TK between the spondylolysis and control groups. For all analyses, the level of significance was set at *p* < 0.05.

## 3. Results

Table 1 presents the overall results of this study. Split-plot ANOVA for LL showed no interaction effect (F(1, 14) = 0.36, *p* = 0.56, partial η^2^ = 0.025). There was a main effect for the group factor, with LL being significantly larger for spondylolysis patients than for the controls (F(1, 14) = 9.1, *p* = 0.009, partial η^2^ = 0.39). There was no main effect of the position factor (F(1, 14) = 3.5, *p* = 0.83, partial η^2^ = 0.20).

Split-plot ANOVA for SS revealed an interaction effect (F(1, 14) = 4.8, *p* = 0.046, partial η^2^ = 0.25). Simple main effects showed that the SS of the control group was significantly larger in the elevation position than during standing (*p* < 0.001), whereas the SS of spondylolysis patients was not significantly different between the standing and elevation positions (*p* = 0.19). In addition, SS was significantly larger in spondylolysis patients than in the controls while standing (*p* = 0. 02), whereas there was no significant difference between spondylolysis patients and controls in the elevation position (*p* = 0.17).

There was no significant difference in TK between the spondylolysis and control groups (*p* = 0.62).

## 4. Discussion

For the alignment of healthy subjects, LL was reported as 44 (range: 14–69) [22], 60 ± 14 [23]; SS was reported as 40.6 (range: 27.2–59.3) [20], 39.9 (range: 21.2–65.9) [24], 39.6 (range: 17.5–63.4) [9], 39 ± 9 [23]; TK was reported as 35 (range: 9–53) [22], 46.3 (range: 23.0–65.9) [9], 38 ± 12 [23]. The control group in this study showed similar results.

In this study, LL was significantly larger in participants with spondylolysis than controls. A previous study [13] reported that an increase in LL may lead to an increase in force on the pars interarticularis with lumbar extension. Therefore, we believe that spondylolysis patients need to achieve improvements in lumbar hyperlordosis alignment in not only standing but also maximal elevation position of both upper limbs, as increased stress on the fracture site may negatively affect prognosis. In addition, we hypothesized that LL would increase during standing in spondylolysis compared to control and that LL would increase with upper limb elevation only in patients with spondylolysis. The effect size of the LL interaction obtained from the results of this study was 0.025, which is small. The reason our results differed from the hypothesis regarding LL may be attributed to the fact that the effect size was overestimated and that the sample size was underestimated. Therefore, we believe that the effect size should be smaller when examining the LL interaction.

In this study, the SS of the control group was significantly larger in the elevated position than in the standing position, while the SS of the spondylolysis participants was not significantly different between the two positions. This suggests that patients with spondylolysis may be characterized by a decreased sacral slope motion with upper limb elevation. In addition, our results showed that the SS was significantly larger in the spondylolysis group than in the control group in standing position, while there was no significant difference between the two groups in the elevation position. Therefore, the decrease in sacral slope movement may be related to the induction of sacral hyper-slope in the standing position. SS is an indicator of the risk of progression from spondylolysis to spondylolisthesis [14]. In addition, decreased sacral slope motion may result in compensation in different joints. Therefore, we believe that individuals with spondylolysis need to first improve the sacral hyper-slope alignment while standing, following which sacral slope movement should be improved. Further, the spondylolysis patients in this study were within the healthy subject range of previous studies [9,20,23,24], but the race and age range differed. In addition, the effect size of the SS interaction obtained from the results of this study was 0.25, which is large. Therefore, we consider the difference between the two groups to be a meaningful and significant difference.

The results of this study showed no significant difference in TK between the two groups. A previous study [17] reported that an increase in TK in standing position limits upper limb elevation; this suggests that TK in standing position may have had less influence on upper limb elevation. However, compensatory movements of the thoracic spine with upper limb elevation may occur in spondylolysis patients and further research is needed.

This study has four major limitations. First, patients with throwing elbow injuries were enrolled as controls. It is difficult to obtain X-ray films in healthy subjects because of exposure to radiation and the medical costs involved. However, we believe that the control was as close to healthy as possible because the elbow injuries generally do not include the spine. In the future, it will be necessary to explore accurate and useful methods in addition to X-ray films. Second, the subjects in this study mostly had L5 fractures. The effects of differences in fracture level and site (bilateral or unilateral) [8] require further investigation. Third, we could not compare baseball players with different athletes. Therefore, it is unclear whether these results are specific to baseball players, and we believe that further research is needed on this. Fourth, this study included adolescents. Spinal alignment has been reported to change with age [25], and we believe that further studies are needed to examine different age-groups of patients with spondylolysis.

## 5. Conclusions

We investigated baseball players with spondylolysis and those without low back pain. This study showed that the LL was significantly larger in spondylolysis patients than in controls. SS increased only in the control group, with the maximal elevation position of both upper limbs. In addition, the SS in the standing position was significantly larger in the spondylolysis group than in the control group, whereas there was no significant difference in the maximal elevation position of both upper limbs. There was no significant difference in TK between patients with spondylolysis and the controls. Therefore, physical therapy for spondylolysis patients should focus on achieving hyperlordosis alignment in the standing and maximal elevation positions of both upper limbs, sacral hyper-slope alignment in the standing position, and decreased sacral slope motion.

## Figures and Tables

**Table 1:** Spinal alignment in spondylolysis patients and controls.

		**Spondylolysis**	**Control**
Lumbar lordosis angle (LL, °)	Standing	36.9 ± 5.1 ^a^	29.0 ± 5.2
	Elevation position	39.9 ± 7.9 ^a^	30.5 ± 4.7
Sacral slope angle (SS, °)	Standing	35.1 ± 6.4 ^b^	26.9 ± 5.3 ^c^
	Elevation position	36.6 ± 5.8	31.8 ± 6.3
Thoracic kyphosis angle (TK, °)	Standing	22.1 ± 5.2	23.4 ± 3.8

^a^: *p* < 0.01, versus control. ^b^: *p* < 0.001, versus control of standing. ^c^: *p* < 0.05, versus control of elevation position.

## Data Availability

All relevant data are within the paper and its Supplementary Materials files.

## Supplementary Materials

The following supporting information can be downloaded at: https://www.mdpi.com/article/10.3390/ijerph20043231/s1, File S1. Data set for all participants.
